# COVID‐19 herd immunity in Lebanon: Challenges and prospects

**DOI:** 10.1002/puh2.167

**Published:** 2024-03-20

**Authors:** Christopher Maatouk, Orestis Germanos, Anna‐Maria Aad, Georges Gandour, Julian M. A. Buban, Ralph Maatouk, Michelle Zeina, Shyam Sundar Budhathoki, Don Eliseo Lucero‐Prisno

**Affiliations:** ^1^ Faculty of Medical Sciences Lebanese University Hadath Lebanon; ^2^ University of Cyprus Medical School Nicosia Cyprus; ^3^ Faculty of Medicine and Medical Sciences University of Balamand Koura Lebanon; ^4^ College of Medicine University of the Philippines Manila Manila Philippines; ^5^ School of Medicine Lebanese American University Byblos Lebanon; ^6^ School of Public Health Imperial College London London UK; ^7^ Department of Global Health and Development London School of Hygiene and Tropical Medicine London UK; ^8^ Faculty of Management and Development Studies University of the Philippines Open University Los Banos Laguna Philippines; ^9^ University of Makati Makati City Philippines

**Keywords:** COVID‐19, herd immunity, Lebanon, vaccination, vaccine hesitancy

## Abstract

COVID‐19 hit Lebanon at the worst time, amid an economic downward spiral and national protests regarding living conditions and political corruption. The first case was found on February 21, 2020, and the first batch of vaccines arrived on March 24, 2021. Although neither natural infection nor mass vaccination truly provided herd immunity, the latter was a more effective way to handle the pandemic, and Lebanon fell short on that path. During the pandemic, a myriad of factors complicated its response to the virus. Thanks to the COVAX program, the country received 1086,720 doses donated and 1626,390 deliveries. All in all, over 5.8 million doses have been administered. A total of 2.74 million people received at least one dose, and 2.4 million had a complete primary vaccination series. However, around 98% of the population were infected with the virus. Issues that stopped the vaccination campaigns include a lack of trust in government officials and news media, leading to false information propagating and remaining unchallenged online. Other factors include the economic collapse, which led to the Lebanese currency losing over 98% of its initial worth. Some Lebanese people might find themselves either unable to reach proper health facilities or unwilling to adopt the narrative pushed by the political elite. Poverty also worsened affected infected individuals’ prognosis and mortality. A bigger emphasis must be put on reaching individuals with disabilities or in low‐income areas, as they were the most affected by the pandemic. Problems these communities face include the lack of funding for special education schools and the lack of accessibility to medical information promoted by the government. Lebanon must learn from the issues that arose during this pandemic and focus on fixing them in advance to prepare for any other health emergency that might turn up in the future.

## Graphical abstract

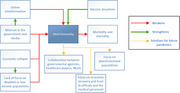

## INTRODUCTION

Lebanon is a Middle Eastern country with a population roughly of 5490,000 [1]. It has recently received negative attention in the international spotlight due to the hyperinflation of the Lebanese pound since 2019 and the Beirut port explosion in 2020. These issues that the country has been repeatedly hit by stem from long‐standing political corruption. Hence, the COVID‐19 pandemic could not have come at a worse time for the country. COVID‐19 became the number one public health concern worldwide as it was categorized as a pandemic on March 11, 2020, 2 months after being reported to the World Health Organization [2]. The first case of COVID‐19 in Lebanon was reported on February 21, 2020. Since then, three waves of infection have struck the country, with daily new cases peaking in August 2020, January 2021, and March 2021 and reaching an all‐time high on February 4, 2022, with 9446 new cases [3]. As of October 25, 2023, there have been more than 1.2 million confirmed cases and 10,947 deaths from COVID‐19 in Lebanon [3]. This study aims to elucidate Lebanon's challenges during the outbreak and how these issues exacerbated this pandemic. We will also discuss the post‐pandemic period to better prepare for any impending pandemics.

## COVID‐19 VACCINATION EFFORTS

The vaccines that the Ministry of Public Health approved include the Pfizer‐BioNTech Comirnaty, Serum Institute of India Covishield (Oxford/AstraZeneca formulation), Sinopharm (Beijing) Covilo, and Gamaleya Sputnik V vaccines [4]. The first batch of the COVID‐19 vaccine received by Lebanon was the Oxford‐AstraZeneca vaccine on March 24, 2021 [5]. Through the COVAX program initiated by the World Health Organization, Lebanon received 1086,720 doses donated and 1626,390 deliveries [6]. According to the WHO, as of December 21, 2023, more than 5.8 million doses have been administered, with 2.74 million people being vaccinated with at least one dose and 2.4 million having had a complete primary series [3]. According to a briefing by the Institute for Health Metrics and Evaluation released on December 15, 2022, around 98% of the Lebanese population has been infected at least once. Vaccination campaigns have virtually come to a halt in 2023 [7].

## HERD IMMUNITY

Herd immunity is a protection phenomenon achieved when a sufficiently large proportion of the population is immune to a contagious disease. An immune population restricts the infectious agent's capacity to spread, thereby limiting infectious exposure to the few who are not immune. Herd immunity has been widely recognized as an essential mechanism for the long‐term control and elimination of certain infectious diseases. It was believed that attaining herd immunity for COVID‐19 could be achieved either by natural infection or mass vaccination. However, noting the wide reach of infection and the vaccination campaign, Lebanon did not achieve herd immunity per se. Nevertheless, as opposed to the natural infection route, the vaccination campaign has decreased the number of severe infections and deaths. A study found that the baseline vaccine efficacies for hospitalization and mortality were 92% and 91%, respectively [8]. Therefore, the lack of herd immunity could cause further deaths and exacerbate underlying diseases in patients who are at risk. Given that the vaccination route is a more effective way of handling the pandemic, Lebanon ought to find solutions to vaccine noncompliance or nonavailability for future pandemics. Should the Lebanese population be unable to do so, a future outbreak could worsen the already present economic hardships for the population. Based on the previously mentioned number of vaccinations and natural infections, Lebanon faced issues that impeded its reliance on the mass vaccination process.

Calculating the percentage of vaccinated people needed to achieve herd immunity requires understanding the dynamics of SARS‐CoV‐2 spread. Integral to such calculations is the basic reproductive number *R*_0_, which is the average number of new individuals a given case will infect during the pathogenic transmission period within a fully susceptible population. As there are no fully susceptible populations, this parameter is theoretical. A more pragmatic metric is the effective reproductive number *R*, where *R *= *R*_0_ multiplied by the percentage of people that can be infected in the population. If *R*_0_ is less than one, the pathogen will tend toward eradication; if *R*_0_ is equal to one, the pathogen will become endemic; and if *R*_0_ is greater than one, the pathogen will tend toward epidemic spread. To achieve herd immunity and take steps toward SARS‐CoV‐2 eradication, *R*_0_ and *R* must be less than one. The effect of vaccines on viral spread can be quantified with a variable *F*, which is the proportion of the population immune from infection. Furthermore, the fraction of the vaccinated population that is not receptive to infection can be represented with the variable *h*. For the COVID‐19 pandemic to trend toward eradication, the percentage of immune and immunized individuals *F* must be high enough so that *F* > [1 − (1/*R*_0_)]/*h*. This equation is valuable in estimating the percentage of the population that must be vaccinated in order to reach the herd immunity threshold [9].

Vaccinating the required number of people to reach the herd immunity threshold depends on their willingness to participate in vaccination programs and requires a significant public effort to reach as many people as possible. To this end, Lebanon launched a public campaign on May 24, 2021 [10]. The campaign consists of three arms: (1) spreading awareness of the importance of the vaccine and encouraging people to register on Lebanon's COVID‐19 vaccination platform, (2) securing necessary medical equipment for hospitals and health centers, and (3) providing 40,000 vaccines for Palestinian refugees in Lebanon. The campaign prioritized the vaccination of the elderly and other vulnerable groups [11].

## CHALLENGES TO ACHIEVING HERD IMMUNITY

At the end of 2019, the Lebanese economy began to deteriorate due to perceived government corruption, which resulted in massive protests and riots. The value of the Lebanese pound has since been in a constant downward spiral, losing over 98% of its previous worth [12]. Hyperinflation ensued, and the Lebanese people had to grapple with both economic hardship and the risk of contracting a virus that could potentially impose a hefty hospital bill or, at the bare minimum, now expensive drugs from the pharmacy. Due to financial stress compounded by rising gas prices, people have limited their transportation to the essentials, such as grocery shopping and pharmacy visits. Hence, should a hospital be out of reach to some, they may not have the means to travel long distances for an optional preventive treatment.

However, financial hardship is not the only hindrance to herd immunity. A growing mistrust of the government has led many people to demand that all current politicians resign and call for early elections. In mid‐January 2022, hundreds of Lebanese people and religious leaders gathered in the capital to protest against receiving the vaccine and especially against making it mandatory. According to these protestors, the government is infringing on their right not to receive the vaccine. A lack of trust in the Lebanese government's ability to handle the pandemic was shown to have exacerbated anxiety [13]. Hence, despite the widely accepted proof of the vaccine's efficacy, some individuals have undergone so much stress due to government policy that they will oppose any action it recommends. In 2021, only 21.4% of Lebanese people were willing to receive the vaccine, whereas 40.9% refused. Women and married people were more likely to refuse the vaccine [14]. A more recent study among Lebanese health workers found similar results regarding sex and added that those who were older and had never been infected were more likely to accept getting vaccinated [15].

Although indirectly related to the pandemic, the dire socioeconomic environment and citizens’ mistrust of the Lebanese government downplay its message about the vaccine. This mistrust was heightened when an employee at Batroun Hospital was found to have given a mixture of salt and water while telling people that it was a Pfizer vaccine shot [16]. Many television channels in Lebanon are also distrusted because nearly all of them have political ties to government parties, creating a lack of trusted information. Therefore, many individuals now rely on news from online sources, including WhatsApp, regardless of evidence. Although no entity can be regarded as the sole truth‐teller, the information circulated online is unchallenged. False information, often embedded with outrageous claims, travels fast, reinforcing people's confirmation bias. Due to people's wariness regarding the government, negative claims about the vaccine that it is endorsing only reinforce the idea of the government's nefariousness. Masks are no longer mandated in public settings, and no lockdowns are being set. Were it not for the issues mentioned, Lebanon could have relied better on the vaccination process, which would have made the pandemic less harmful to its public's health.

## POST‐PANDEMIC PERIOD

The aftermath of the pandemic in Lebanon has raised serious worries about trust in government institutions and the economy's health. Notably, the healthcare sector has suffered substantial consequences, leading to a decreased propagation of medical information that could have been necessary for individuals. Going forward, the Lebanese government must focus more on marginalized populations, most notably people with disabilities, as they were the most affected by the deficits mentioned. During the pandemic, although the government posted online videos to teach the public about safety measures concerning the pandemic, it did not, for the most part, make these videos accessible to those with disabilities, for example, deaf or blind individuals. The rollout of the COVID‐19 vaccine also did not consider checking for those with disabilities. Additionally, special education schools did not receive the funding the government owed them [17, 18]. Being aware of these shortfalls will allow the Lebanese government to handle the next pandemic better.

The impact of the economic crisis on Lebanon's healthcare system also includes the loss of frontline healthcare professionals as well as the government's diminishing allocation for healthcare due to fiscal constraints [19]. These difficulties resonate across the healthcare system and jeopardize the availability and quality of critical medical treatments. Furthermore, the compounding crises involving people with disabilities during the pandemic highlight their increased vulnerability and exclusion from mainstream education and healthcare systems [20]. The combination of economic insecurity and healthcare inequality emphasizes the importance of coordinated measures to address systemic deficiencies and strengthen resilience in Lebanon's healthcare ecosystem. Another study focusing on the obstacles faced by Lebanon's health system in its reaction to COVID‐19 showed gaps in care provision and complications in decision‐making dynamics [21]. Such findings highlight the crucial need for coordinated initiatives to strengthen healthcare communication channels and protect public health infrastructure against future disasters.

Given these hurdles, improving Lebanon's healthcare system's preparedness and capacity is critical to successfully navigating the post‐pandemic scenario. Concerted efforts are needed to address the numerous aspects of healthcare delivery, including improved communication techniques, increased workforce resilience, and targeted interventions to reduce inequities among vulnerable populations. Collaboration among governmental agencies, healthcare players, NGOs, and civil society is required to build a resilient healthcare infrastructure capable of withstanding future crises while protecting Lebanon's population's health and well‐being. If the country does not address the issues at hand, another health crisis might cause more devastating outcomes than this last one. The virus is currently in the background in most people's minds. However, trust in authority figures, including medical personnel, must be restored for Lebanon to rise from all the hurdles it faces. The country must also fix the economic collapse it is facing if it wants to afford to battle another pathogen.

## CONCLUSION

In conclusion, many variables have complicated Lebanon's path to herd immunity against COVID‐19. The obstacles of a frail healthcare system, economic insecurity, and vaccine delivery challenges have tested the nation's resilience. Despite these limitations, there have been some notable accomplishments and many lessons to be learned. It is impossible to overestimate the necessity of a well‐coordinated and effective public health response. Lebanon's experience emphasizes the importance of taking proactive measures to restrict the spread of a virus, such as timely testing, contact tracing, and clear communication tactics. The epidemic has exposed flaws in the healthcare infrastructure, encouraging the country to invest in improving its medical capabilities in preparation for future crises. Vaccine equity emerges as a critical learning point. It is critical to ensure that vaccines reach all parts of the population, regardless of socioeconomic status or disability, to achieve widespread immunity. International collaboration and assistance can be critical in overcoming resource constraints and increasing vaccine accessibility. Despite Lebanon's evident community engagement and awareness, the broader issues must be addressed, including the economic collapse and the distrust in the political class and news media. This way, a public health campaign can effectively promote shared responsibility and debunk false information. Lebanon's road toward herd immunity serves as a reminder that global health concerns necessitate a united stance. Solidarity, both inside and across borders, is critical for overcoming the complications imposed by pandemics. The lessons learned in Lebanon highlight the need to develop resilient healthcare systems, encourage equity, and cultivate a spirit of cooperation in the face of adversity as the globe adjusts for potential future health crises.

## AUTHOR CONTRIBUTIONS

Don Eliseo Lucero‐Prisno III conceptualized the paper. Christopher Maatouk, Orestis Germanos, Anna‐Maria Aad, and Georges Gandour contributed to collecting data and information and writing the first draft. Ralph Maatouk and Julian M. A. Buban revised the second draft. Michelle Zeina contributed to the revision of the paper. Everyone approved the final draft.

## CONFLICT OF INTEREST STATEMENT

Don Eliseo Lucero‐Prisno III is Editor‐in‐Chief of the journal and coauthor of this article. Shyam Sundar Budhathoki is an Editorial Board member of Public Health Challenges and a coauthor of this article. To minimize bias, they were excluded from all editorial decision‐making related to the acceptance of this article for publication.

## Data Availability

Data sharing is not applicable to this article as no new data were created or analyzed in this study.
